# Effectiveness of a self-management training for patients with chronic and treatment resistant anxiety or depressive disorders on quality of life, symptoms, and empowerment: results of a randomized controlled trial

**DOI:** 10.1186/s12888-019-2013-y

**Published:** 2019-01-28

**Authors:** Maringa H. H. Zoun, Bauke Koekkoek, Henny Sinnema, Christina M. van der Feltz-Cornelis, Anton J. L. M. van Balkom, Aart H. Schene, Filip Smit, Jan Spijker

**Affiliations:** 1 Behavioural Science Institute, Radboud University Medical Center, PO Box 9104, 6500 HE Nijmegen, The Netherlands; 2 Pro Persona Mental Health Care, Wolfheze 2, 6874 BE, Wolfheze, The Netherlands; 3 Pro Persona Mental Health Care, Wagnerlaan 2, 6815, AG Arnhem, The Netherlands; 4 Research Group for Social Psychiatry and Mental Health Nursing, HAN University of Applied Science, PO Box 6960, 6503 GL Nijmegen, The Netherlands; 5 Institute for Nursing Studies, HU University of Applied Sciences, Heidelberglaan 7, 3584 CS Utrecht, The Netherlands; 6 Top Clinical Centre for Body, Mind and Health, GGZ Breburg, Lage Witsiebaan 4, 5042 DA Tilburg, The Netherlands; 7 Department of Health Sciences, HYMS, York Biomedical Research Institute, University of York, Room ARRC/204, Area 4, ARRC Building, YO10 5DD Yorkshire Heslington, UK; 8 Department of Psychiatry and Amsterdam Public Health Research Institute, VU University Medical Centre and GGZ inGeest, Oldenaller 1, 1081 HJ Amsterdam, The Netherlands; 9 Department of Psychiatry, Radboud University Medical Center, PO Box 9101, 6500 HB Nijmegen, The Netherlands; 10 Donders Institute for Brain, Cognition and Behavior, Radboud University, Montessorilaan 3, 6525 HR, Nijmegen, The Netherlands; 11 Department of Clinical, Neuro and Developmental Psychology, VU University Medical Centre, Van der Boechorststraat 1, 1081 BT Amsterdam, The Netherlands; 12 Department of Epidemiology and Biostatistics, VU University Medical Centre, Van der Boechorststraat 1, 1081 BT Amsterdam, The Netherlands; 13 Trimbos Institute (Netherlands Institute of Mental Health and Addiction), PO Box 725, 3500 AS Utrecht, The Netherlands

**Keywords:** Anxiety, Depression, Chronic, Treatment resistant, Self-management, Randomized controlled trial, Specialized outpatient mental health care, Primary care

## Abstract

**Background:**

Anxiety and depressive disorders are common mental disorders. A substantial part of patients does not achieve symptomatic remission after treatment in specialized services. Current care as usual (CAU) for these patients consists of long-term supportive contacts. Termination of CAU is often not considered to be an option due to persistent symptoms, a low level of functioning, and the absence of further treatment options. A new intervention, ZemCAD, offers a program focused on rehabilitation and self-management, followed by referral back to primary care.

**Methods:**

This multicenter randomized controlled trial was carried out in twelve specialized outpatient mental health care services in the Netherlands. Consenting and eligible patients were invited for the MINI interview and the baseline questionnaire. Assessments were done at 6 (T1), 12 (T2) and 18 (T3) months post baseline. We used linear mixed model analysis (LMM) to ascertain the effectiveness of the ZemCAD group relative to the CAU group on quality of life, symptom severity and empowerment.

**Results:**

In total 141 patients were included. The results at 18-month follow-up regarding to quality of life and symptom severity, showed no significant differences between the ZemCAD group and the CAU group, except on the ‘social relationships’-domain (*d* = 0.37). With regard to empowerment a significant difference between both groups was observed in the total empowerment score and one empowerment dimension (*d* = 0.45 and *d* = 0.39, respectively). After the ZemCAD intervention, more patients went from specialized outpatient mental health services back to a less specialized health care setting with less intensive treatment, such as primary care.

**Conclusion:**

The findings in this study suggest that patients with chronic and treatment-resistant anxiety and depression using the ZemCAD intervention improve on empowerment but not on symptom severity or quality of life. Since little is known about the effects of rehabilitation and self-management in patients with chronic and treatment resistant anxiety and depressive disorders, this is a first attempt to provide a proof-of-concept study in this under-researched but important field.

**Trial registration:**

Netherlands Trial Register: NTR3335, registered 7 March 2012.

## Background

Anxiety and depressive disorders are common mental disorders. Lifetime prevalence in the Netherlands, according to NEMESIS-2 (Netherlands Mental Health Survey and Incidence Study-2), is 19.6% for anxiety disorders, and 18.7% for depressive disorders [1, 2], not much different from other developed countries. In specialized outpatient mental health services, anxiety and depressive disorders are also common: 22.2% of outpatients have an anxiety disorder and 37.2% a depressive disorder [2]. Treatment for these patients generally is provided in accordance with (inter-)national multidisciplinary guidelines [3–5]. However, a substantial part of these patients does not achieve symptomatic remission after treatment in these specialized services, resulting in so-called treatment resistance [6] and a chronic course. The estimated chronicity for anxiety disorders is 41.9, and 24.5% for depressive disorders [7]. Chronic and treatment resistant anxiety and depressive disorders are associated with more intense suffering, increased risk of suicide, and decreased social functioning [8, 9].

Current care as usual (CAU) for patients with chronic and treatment-resistant anxiety or depressive disorders in many countries consists of long-term supportive contacts with a community psychiatric nurse, combined with pharmacological management by a psychiatrist. Termination of CAU is often not considered to be an option due to persistent symptoms, a low level of functioning, and the absence of further treatment options. Data on the effectiveness on this CAU is lacking [10]. In order to offer these patients a different perspective, a psychosocial rehabilitation approach focused on specific goals could be more suitable [11, 12]. However, research on the feasibility and effectiveness of rehabilitation approaches for chronic and treatment resistant anxiety and depressive disorders in specialized outpatient mental health care is not available.

An increasingly important element in rehabilitation programs, is a focus on self-management. In long-term health problems, self-management is a method to focus on enhancement of patients’ autonomy and responsibility and on the restoration of functioning – less on the reduction of symptoms. Self-management refers to “the training, skill acquisition, and interventions through which patients who suffer from a disease or chronic condition may take care of themselves and manage their illnesses” [13]. Self-management is considered essential in managing a chronic disease. We can differentiate three levels of self-management: 1) the shift from a compliant model to a participation model, where responsibilities and management shifts from professional to patient, 2) the ability to handle the chronic disease, and 3) self-management as intervention to train the patient to handle a chronic disease properly [14]. Self-management leads to patients’ empowerment, and it may also contribute to quality of life, which is known to be lower in people with more symptoms [15–19]. Some research is done into helpful self-management strategies to cope with enduring depression from the patients’ point of view [20–22], but, to our knowledge, little is known about the effects of rehabilitation and self-management on quality of life, symptom severity, and empowerment in patients with chronic and treatment resistant anxiety and depressive disorders.

In addition to self-management, empowerment as a concept in mental health care has been receiving growing attention in the last years. The concept is defined in many ways, but “a distinction can be made between empowerment at the individual and the collective level. At the individual level, empowerment is about processes in which the person rediscovers his identity and self-esteem and takes his life in his own hands. At the collective level, it is about the contribution by people with lived experience to the organisation and practice of mental health care and society” [23].

There are, to our knowledge, hardly any evidence-based treatment interventions for patients with chronic and treatment-resistant anxiety and depressive disorders. Due to the transition of the Dutch health care system where the aim is to refer chronic patients to less intensive levels of care when possible, we need to make cost-effective use of resources. Therefore a new intervention is developed with focus on self-management combined with the transition from specialized outpatient mental health care to primary care. The new intervention, ZemCAD (English: SemCAD; Self-management for Chronic Anxiety and Depression), was developed for patients with chronic and treatment resistant anxiety or depressive disorders in specialized outpatient mental health care. Treatment resistant patients in this study have been described as patients who had received at least one psychological treatment and at least three medication steps according to the national multidisciplinary guidelines on anxiety and depressive disorders. The intervention is provided by a trained professional (usually a community psychiatric nurse). In this randomized controlled trial [24], the effects of the ZemCAD intervention on quality of life, symptom severity, and empowerment compared to CAU are evaluated.

We had two research questions. First, compared to CAU, what is the effectiveness of the ZemCAD intervention on quality of life, symptom severity and empowerment? Second, how successful was the referral of patients included in the ZemCAD intervention from specialized outpatient mental health services to less specialized health care settings, such as primary care?

## Methods

### Design and participants

This multicenter randomized controlled trial in two parallel groups was carried out in twelve specialized outpatient mental health care services in the Netherlands. These twelve services offered outpatient treatment interventions in accordance with the (inter-)national multidisciplinary guidelines, and prolonged treatment for patients with chronic and treatment resistant anxiety or depressive disorders.

Participants had an anxiety or depressive disorder according to the DSM-IV, were over 18 years old, received treatment for at least two years in specialized outpatient mental health care, had received at least one psychological treatment and at least three medication steps according to the national multidisciplinary guidelines on anxiety and depressive disorders [25]. They were regarded as treatment-resistant by their clinicians, meaning that prolonged treatment in a specialized outpatient mental health service according to the professional is unlikely to improve clinical outcomes. They had supportive contacts with a community psychiatric nurse. All gave written informed consent to participate in the study. Patients were excluded from participation if they had a life-threatening medical condition, dementia, psychotic or bipolar disorder, alcohol or drugs dependence, had cognitive problems or indications for low IQ or were not fluent in the Dutch language.

First we asked clinicians to select patients (on a form) who could meet our inclusion criteria. Then we checked the forms. If patients did not fully meet the inclusion criteria, we consulted with the clinician. In some cases we considered a patient who did not fully meet the inclusion criteria suitable to participate. In this case there was a lifetime diagnosis, a low level of functioning, and persistent symptoms. All patients that were eligible for participating in the ZemCAD study were asked to participate by their clinician, who informed the patient about the study and provided an information letter to take home. If the patient was interested and consented to participate, the signed informed consent was sent to the coordinating research centre. Then patients were invited for the Mini-International Neuropsychiatric Interview (MINI interview) to formally check if the current DSM-IV disorders were present. The MINI is a semi-structured and well-validated diagnostic interview to establish psychiatric disorders according to the DSM-IV [26]. The MINI was administered at the mental health service providing the treatment of the patient. After inclusion, patients received the baseline questionnaire.

After completing the baseline questionnaire, the patient was allocated to the ZemCAD group or the CAU group, using a randomization schedule designed by an independent statistician. To evenly distribute ZemCAD/CAU across community psychiatric nurses, block randomization (block size of four) was used. After baseline (T0) assessments were done at 6 (T1), 12 (T2) and 18 (T3) months post baseline. All participants in the ZemCAD group started the intervention after T0 (baseline). The intervention has a duration of twenty-six weeks. So T1 is a post-treatment assessment. T2 and T3 are follow-up assessments. The questionnaires (except for the MINI interview) were completed over the internet. If a patient had no access to the internet, the questionnaires were completed on paper.

In the intervention group (ZemCAD) treatment integrity was assessed for each participating patient using a checklist that the community psychiatric nurse completes at the end of each treatment session. In this checklist the nurse could indicate on which points the treatment differs from the ZemCAD protocol. Patients who were allocated to the CAU group often continued receiving care from their current community psychiatric nurse. Since there is no clear treatment guideline for CAU, treatment integrity was not assessed in the control group.

Patients were informed that participation in the study was voluntary and that they could withdraw from the study at any time. The privacy of the participants was guaranteed by anonymizing the data. This study has been approved by the Institutional Review Board of the University Medical Center Utrecht for all participating sites (NL33674.097.10, registration number 10.218).

### Intervention

The ZemCAD intervention is a treatment protocol for patients with chronic and treatment-resistant anxiety or depressive disorders, and directed at rehabilitation and self-management. It is based on an existing treatment protocol for patients with chronic depression [27], adapted for patients with chronic and treatment resistant anxiety or depressive disorders [28]. The ZemCAD intervention was carried out by 29 trained professionals (usually community psychiatric nurses) in twelve participating specialized outpatient mental health care services in the Netherlands, and at the end followed by referral back to primary care.

Patients receiving the ZemCAD intervention were allocated to a new and trained professional. This clearly marked the transition from CAU to ZemCAD for both patient and professional. The training of the participating professionals in the ZemCAD group consisted of a two-day course. A prerequisite was that the professionals study the intervention in advance. The two-day course was provided by an expert and trainer in cognitive behavioral therapy and motivational skills for anxiety and depressive disorders. The training combined self-study, lectures, assignments, and group discussions. During the study, three additional booster sessions were given. In addition, in each mental health service monthly booster sessions for the professionals were scheduled.

The intervention consists of thirteen sessions over twenty-six weeks. First, patients and their families were educated about the nature of their chronic disorder, about suicidality and crises, and they learn how to cope with these conditions and events. An action plan to re-establish social contacts and improve daily living activities are examples of parts of the intervention.

The ZemCAD intervention consists of three parts. The first part is an introduction phase of three weeks with weekly sessions. The goals for the professionals are to get acquainted with the patient and family, and explain the treatment. Patients make an individual treatment plan, identify symptoms and daily activities, keep a log of symptoms, and learn how they can accept lifestyle changes due to having a chronic disease. The second part is a coaching and treatment phase of fourteen weeks with sessions every second week. Patients start again engaging in social activities, they are stimulated to maintain a daily structure, and learn to use general problem solving skills to cope with their chronic disease. The third part is the final phase of nine weeks with sessions every three weeks. Topics are to make an action plan on how to deal with crisis situations and to further practice with earlier mentioned skills. Finally, patients are referred to primary care. Every primary care practice is asked to select a mental health professional who works in close collaboration with the general practitioner and actively monitors functioning of the patient. After referral to primary care, the general practitioner is responsible for the prescription of medication. Both mental health professional in primary care and general practitioner have easy access to specialized outpatient mental health services for liaison consultation if required.

### Control condition

Patients who were allocated to the CAU group continued to receive specialized outpatient mental health care, which usually consisted of long-term supportive contacts with a community psychiatric nurse, and pharmacological management by a psychiatrist. Apart from that, some patients had contacts with a psychologist or a nurse practitioner, or just a psychiatrist. But in all cases it concerned supportive treatment. There was no other treatment at that time, such as cognitive behavioural therapy. CAU may also involve termination of treatment in specialized outpatient mental health care and referral back to primary care. In the ZemCAD intervention, however, this referral is planned within the intervention and therefore anticipated, while in the CAU group the referral and termination of the treatment only occurs when indicated.

### Measurements and outcomes

At baseline demographics, MINI diagnosis, quality of life, symptom severity, and empowerment were measured. Follow-up measurements were on quality of life, symptom severity, and empowerment. These measurements took place 6, 12, and 18 months after baseline. Since referral back to primary care is part of the ZemCAD intervention, we conducted an additional questionnaire after finishing the intervention. Among other questions, we asked patients where they currently received treatment (specialized outpatient mental health care or primary care).

#### Quality of life (main outcome)

Quality of life was measured with the World Health Organization Quality of Life instrument, Brief version (WHOQOL-BREF) [29, 30]. The WHOQOL-BREF is a 26-item version of the WHOQOL-100 assessment. It produces a quality of life profile with four domain scores with good established psychometric properties: physical health (Cronbach’s α = 0.82), psychological health (Cronbach’s α = 0.81), social relationships (Cronbach’s α = 0.68), and environment (Cronbach’s α = 0.80) [29, 30]. The scores on the WHOQOL-BREF are scaled in a positive direction (i.e. higher scores mean higher quality of life).

#### Anxiety severity

Anxiety severity was measured with the Beck Anxiety Inventory (BAI), with good established psychometric properties (Cronbach’s α = 0.92) [31]. It is a validated self-rated questionnaire to assess the severity of anxiety symptoms. The inventory consists of 21 items. The scores on the BAI are scaled in a negative direction (i.e. lower scores mean less symptom severity).

#### Depression severity

Depression severity was measured with the Patient Health Questionnaire-9 (PHQ-9), a validated self-rated questionnaire to assess the severity of depression symptoms with good established psychometric properties (Cronbach’s α = 0.86–0.89) [32, 33]. It includes the 9 symptoms of the DSM-IV depressive episode. A higher PHQ score means higher severity.

#### Empowerment

Empowerment is assessed using the Netherlands Empowerment List (NEL), with good established psychometric properties (Cronbach’s α = 0.94) [23, 34]. It is a 40-item self-report questionnaire to assess empowerment. Respondents indicate the extent to which they agree or disagree with the statements on a five-point scale. The NEL produces an overall empowerment score, and in addition it is possible to derive six dimension scores: professional help, social support, confidence and purpose, connectedness, self-management, and caring community. The scores on the NEL are scaled in a positive direction (i.e. higher scores mean more empowerment).

### Analyses

Descriptive statistics were used for presenting the demographics of the sample. All outcome analyses were conducted in agreement with the intention-to-treat (ITT) principle, as required by the CONSORT statement [35]. The estimated marginal means were calculated to see the mean response for each outcome variable over time. We used linear mixed model analysis (LMM) to ascertain the relative effectiveness of the ZemCAD group relative to the CAU group on the WHOQOL-BREF, BAI, PHQ-9, and NEL. If differences in baseline data were present, we adjusted for relevant baseline characteristics, by including baseline variables as covariates in the model. The linear mixed models were carried out in two steps. First we entered the treatment variable (ZemCAD vs CAU) and the covariates for which adjustments were needed into the model as fixed effects. As random effects, we entered the subject_ID variable into the model. The covariance structure was ‘unstructured’, and the method was ‘residual maximum likelihood’ (reml), which offers better estimates in smaller samples. When the main effect of the condition was statistically significant, we carried out the second step to see how effects developed over time. To that end, we also modelled interactions of the treatment with time. The results were considered significant at *p* < 0.05. The standardized difference between two groups, Cohen’s *d,* was computed from the value of the t-test of the differences between the two groups: *d* = 2 t / √(df) [36]. In the equation ‘df’ is the degrees of freedom for the t-test. All analyses were conducted in SPSS 22 and Stata 14.2.

### Recruitment and patient characteristics

**Fig. 1:**
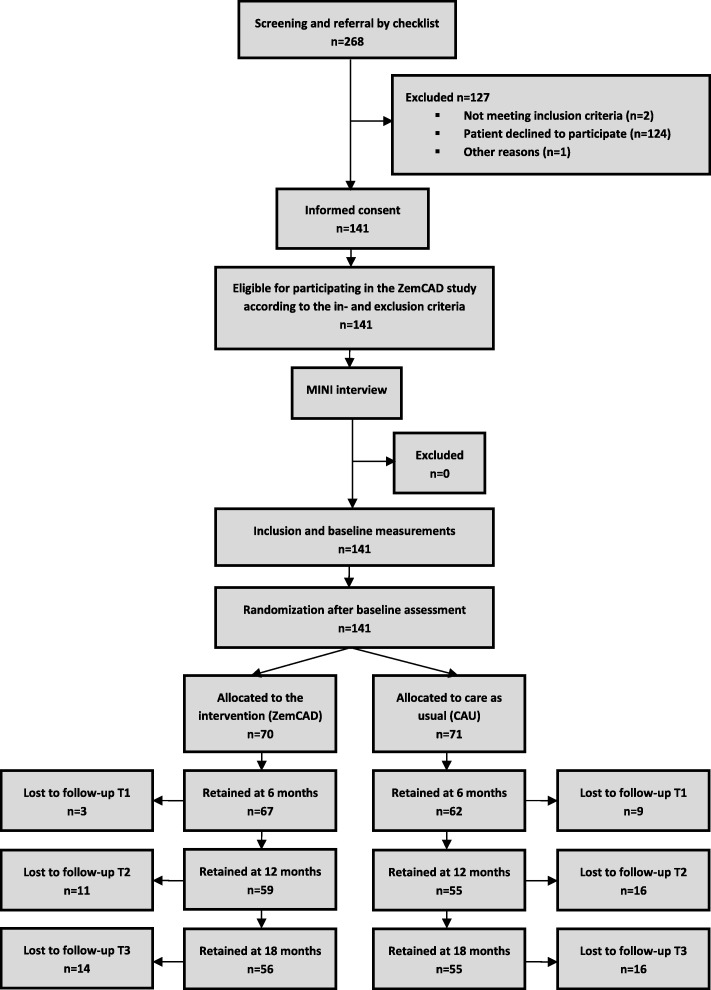
Flowchart of participants

In total 268 patients were signed up by their mental health care professional to participate in the ZemCAD study, of whom 141 patients were included. 70 patients were allocated to the ZemCAD group, 71 patients were allocated to the CAU group. Almost all of the 127 exclusions were because of the patient’s unwillingness to participate in the research (124). Two patients were excluded because of a psychotic or bipolar disorder. The patient flow through the trial is depicted in Fig. 1.

## Results

### Baseline characteristics

Mean age of the study sample was 47.5 ± 8.3 years, 66.0% was female. According to the MINI interview, 17.0% had an anxiety disorder, 26.2% a depressive disorder, 44.0% had both a depressive and an anxiety disorder, and 12.8% had no current disorder. The mean age at which patients first experienced anxiety or depressive symptoms was 26.6 ± 13.19 years. At baseline, clinically relevant differences were found between the ZemCAD and CAU groups on ‘partner status’ and ‘duration of current symptoms’. There were more singles in the CAU group and also patients had the longest duration of current symptoms in the CAU group. The mean duration of current symptoms was 4.79 ± 7.14 years. There were no clinically relevant differences found on quality of life, symptom severity, and empowerment. The BAI scores are classified as minimal anxiety (0 to 9), mild anxiety (10 to 16), moderate anxiety (17 to 29), and severe anxiety (30 to 63). The PHQ-9 scores are classified as minimal depression (0 to 4), mild depression (5 to 9), moderate depression (10 to 14), moderately severe depression (15 to 19), and severe depression (20 to 27). If we look at the average score on the BAI and PHQ-9 measurements in our sample, we could describe the participants as severely ill. An overview of baseline characteristics is provided in Table 1.

**Table 1:** Baseline characteristics of the participants in the intervention (ZemCAD) and control (CAU) groups (*n* = 141)

	ZemCAD (*n* = 70)	Control (*n* = 71)
Gender, n (%)		
Female	46 (65.70)	47 (66.20)
Male	24 (34.30)	24 (33.80)
Age (years)		
Mean (SD)	47.03 (7.68)	47.87 (8.90)
Range	28–63	27–63
Education, n (%)		
Low	34 (50.00)	38 (53.50)
Intermediate	12 (17.60)	16 (22.50)
High	22 (32.40)	17 (23.90)
Employment, n (%)		
Paid	14 (20.60)	13 (18.30)
No paid	54 (79.40)	58 (81.70)
Nationality, n (%)		
Dutch	59 (84.30)	59 (83.10)
Other	11 (15.70)	12 (16.90)
Partner status, n (%)		
Single	26 (37.10)	39 (54.90)
Living with partner	44 (62.90)	32 (45.10)
Age of first onset, M (SD)	27.57 (12.22)	24.97 (14.07)
Duration of current symptoms (years), M (SD)	3.59 (5.90)	5.99 (8.06)
Diagnosis according to MINI^a^, n (%)		
Anxiety	12 (17.10)	12 (16.90)
Depression	16 (22.90)	21 (29.60)
Both anxiety and depression	29 (41.40)	33 (46.50)
No current anxiety or depression	13 (18.60)	5 (7.00)
Quality of life (WHOQOL-BREF^b^), M (SD)	73.49 (15.34)	72.03 (13.11)
Anxiety (BAI^c^), M (SD)	43.82 (13.57)	45.28 (11.27)
Depression (PHQ-9^d^), M (SD)	22.51 (6.42)	23.18 (6.58)
Empowerment (NEL^e^), M (SD)	121.88 (20.46)	121.48 (16.57)

^a^MINI = MINI International Neuropsychiatric Interview

^b^*WHOQOL-BREF* World Health Organisation Quality of Life, Brief version

^c^*BAI* Beck Anxiety Inventory

^d^*PHQ-9* Patient Health Questionnaire

^e^*NEL* Netherlands Empowerment List

### Effectiveness of the ZemCAD intervention

An overview of linear mixed model analyses on quality of life, anxiety and depressive symptom severity, and empowerment is presented in Table 2 where unadjusted and baseline adjusted effects are presented as the between-group difference in the mean change over all follow-ups simultaneously. The results of these analyses showed that quality of life, measured with the WHOQOL-BREF was not significantly different between both conditions, except on the ‘social relationships’-domain in favour of the ZemCAD intervention (*P* = 0.041), with a medium effect size (*d* = 0.37) in the baseline adjusted analysis. Symptom severity, measured with the BAI and PHQ-9, was not statistically different. Empowerment, measured with the NEL, showed a significant difference between conditions in favour of the ZemCAD intervention (*P* = 0.013) with a medium effect size (*d* = 0.45). When looking at the distinct empowerment dimensions a significant difference is observed between the conditions in favour of the ZemCAD intervention in the ‘caring community’-dimension (*P* = 0.030), with a medium effect size (*d* = 0.39). In the ‘confidence and purpose’-dimension we found a significant difference (*P* = 0.035) in the unadjusted model and a trend towards significance (*P* = 0.056) in the adjusted model in favour of the ZemCAD intervention.

**Table 2:** Estimated differences in mean change of outcomes (unadjusted and adjusted)^a^ between baseline and 18-month follow-up for the intervention group (ZemCAD) versus the control group (CAU)

Outcome		Estimate of mean difference (95% CI)	*t (df)*	*P*	Cohen’s *d*^b^
Quality of life (WHOQOL-BREF^c^)					
Total score	unadjusted model	2.40 (− 0.23 to 5.03)	1.81 (128)	0.073	0.31
	adjusted model	2.42 (− 0.27 to 5.11)	1.78 (129)	0.077	
Domain 1;	unadjusted model	0.95 (− 0.06 to 1.95)	1.87 (127)	0.064	0.29
Physical health	adjusted model	0.86 (−0.17 to 1.89)	1.65 (127)	0.101	
Domain 2;	unadjusted model	0.68 (− 0.07 to 1.42)	1.78 (126)	0.077	0.32
Psychological	adjusted model	0.71 (− 0.07 to 1.48)	1.81 (125)	0.074	
Domain 3;	unadjusted model	0.39 (− 0.03 to 0.81)	1.82 (122)	0.072	0.3
Social relationships	adjusted model	0.46 (0.02 to 0.89)	2.07 (122)	0.041*	
Domain 4;	unadjusted model	0.20 (− 0.85 to 1.25)	0.38 (129)	0.704	0.07
Environment	adjusted model	0.22 (− 0.84 to 1.28)	0.41 (129)	0.685	
Anxiety (BAI^d^)					
Total score	unadjusted model	−0.56 (− 3.11 to 1.99)	−0.44 (117)	0.663	−0.07
	adjusted model	−0.53 (− 3.17 to 2.11)	−0.40 (117)	0.693	
Depression (PHQ-9^e^)					
Total score	unadjusted model	0.06 (− 1.46 to 1.58)	0.08 (126)	0.936	0.01
	adjusted model	0.06 (− 1.51 to 1.63)	0.08 (125)	0.940	
Empowerment (NEL^f^)					
Total score	unadjusted model	5.00 (1.20 to 8.80)	2.60 (127)	0.010*	0.45
	adjusted model	5.00 (1.09 to 8.92)	2.53 (126)	0.013*	
Dimension 1;	unadjusted model	0.64 (− 0.47 to 1.76)	1.14 (129)	0.255	0.24
Professional help	adjusted model	0.76 (−0.38 to 1.90)	1.33 (128)	0.187	
Dimension 2;	unadjusted model	0.80 (−0.29 to 1.90)	1.46 (125)	0.148	0.20
Social support	adjusted model	0.61 (−0.50 to 1.73)	1.09 (124)	0.278	
Dimension 3;	unadjusted model	1.78 (0.13 to 3.42)	2.13 (126)	0.035*	0.35
Confidence and purpose	adjusted model	1.65 (− 0.04 to 3.35)	1.93 (124)	0.056	
Dimension 4;	unadjusted model	0.80 (− 0.14 to 1.74)	1.68 (128)	0.096	0.27
Connectedness	adjusted model	0.75 (− 0.21 to 1.72)	1.54 (128)	0.125	
Dimension 5;	unadjusted model	0.30 (− 0.38 to 0.99)	0.88 (127)	0.380	0.15
Self-management	adjusted model	0.30 (− 0.40 to 1.01)	0.85 (127)	0.396	
Dimension 6;	unadjusted model	0.86 (0.05 to 1.68)	2.09 (123)	0.038*	0.39
Caring community	adjusted model	0.93 (0.09 to 1.77)	2.19 (123)	0.030*	

*Significant difference

^a^Unadjusted model: crude association. Adjusted model: adjusted for ‘partner status’ and ‘duration of current symptoms’

^b^*d* = 2 t / √(df)

^c^*WHOQOL-BREF* World Health Organisation Quality of Life, Brief version

^d^*BAI* Beck Anxiety Inventory

^e^*PHQ-9* Patient Health Questionnaire

^f^*NEL* Netherlands Empowerment List

### How effects developed over time

In addition to the estimated differences in mean change of outcomes between baseline and 18-month follow-up for the intervention group versus the control group (overall intervention effect), we also looked at how effects developed over time. An overview of the estimated marginal means (adjusted for ‘partner status’ and ‘duration of current symptoms’) for all outcomes at baseline, and 6, 12 and 18-month follow-up are presented in Appendix 1. In symptom severity we found no significant differences between conditions. In quality of life, we found a significant difference at 12-month follow-up in favour of the ZemCAD group (*P* = 0.025). Also in empowerment we found significant differences at 6-month (*P* = 0.034) and 12-month (*P* = 0.016) follow-up, both in favour of the ZemCAD group. In both quality of life and empowerment the significant effects do not remain at 18-month follow-up. Because few significant differences were found in the overall intervention effect, we only show the total scores of the questionnaires. An overview on how effects developed over time is provided in Table 3. For more details on how the effects developed over time, including the domains of the WHOQOL-BREF and the dimensions of the NEL, see Appendix 2.

**Table 3:** Estimated differences^a^ in change of outcome in the intervention group over follow-ups at 6, 12 and 18 months

Outcome		Estimate of mean difference (95% CI)	*t (df)*	*P*
Quality of life (WHOQOL-BREF^b^)				
Total score	6 months (T1)	0.69 (− 2.56 to 3.93)	0.42 (354)	0.678
	12 months (T2)	3.88 (0.49 to 7.27)	2.25 (356)	0.025*
	18 months (T3)	1.89 (− 1.53 to 5.31)	1.09 (356)	0.279
Anxiety (BAI^c^)				
Total score	6 months (T1)	0.88 (− 2.37 to 4.12)	0.53 (352)	0.595
	12 months (T2)	−1.31 (− 4.72 to 2.10)	−0.76 (355)	0.451
	18 months (T3)	−0.27 (− 3.70 to 3.16)	−0.16 (355)	0.875
Depression (PHQ-9^d^)				
Total score	6 months (T1)	0.09 (− 1.74 to 1.92)	0.10 (354)	0.921
	12 months (T2)	0.09 (−1.82 to 2.01)	0.10 (358)	0.923
	18 months (T3)	1.14 (− 0.79 to 3.08)	1.16 (358)	0.247
Empowerment (NEL^e^)				
Total score	6 months (T1)	5.41 (0.42 to 10.40)	2.13 (353)	0.034*
	12 months (T2)	6.39 (1.19 to 11.59)	2.42 (355)	0.016*
	18 months (T3)	0.00 (− 5.24 to 5.25)	0.00 (355)	0.999

*Significant difference (of the ‘treatment × time’ effect relative to the control condition at baseline)

^a^All estimates are adjusted for ‘partner status’ and ‘duration of current symptoms’

^b^*WHOQOL-BREF* World Health Organisation Quality of Life, Brief version

^c^*BAI* Beck Anxiety Inventory

^d^*PHQ-9* Patient Health Questionnaire

^e^*NEL* Netherlands Empowerment List

### Change of care setting

After finishing the ZemCAD intervention, we asked patients where they currently received treatment (specialized outpatient mental health care or less specialized mental health care, such as primary care). The response was 70.9% (*n* = 100). In the ZemCAD group 44.0% of patients, and in the CAU group 62.0%, was still receiving treatment in specialized outpatient mental health care (*P* = 0.071). As regards to treatment in primary care, 36.7% of patients in the ZemCAD group, and 28.6% in the CAU group, was receiving treatment there (*P* = 0.389). Then 77.8% of patients in the ZemCAD group, and 66.7% of patients in the CAU group perceived the treatment in primary care as ‘good’ (*P* = 0.495).

### Treatment integrity

Treatment integrity could be assessed in 60 patients in the ZemCAD group (out of 70) using a checklist that the trained professional completed at the end of each treatment session. In total 19 (41.4%) of the patients did not finish all sessions of the ZemCAD intervention. The treatment integrity ‘response and proper protocol execution’ is provided in Table 4.

**Table 4:** Treatment integrity; response and proper protocol execution

Sessions	Response % (n)	Proper protocol execution % (n)
Session 1	85.7 (60)	
a: treatment and research discussed?		98.3 (59)
b: explanation about the intervention?		98.3 (59)
c: symptom severity established?		51.7 (31)
d: treatment agreement prepared?		74.6 (44)
e: appointment series made?		63.3 (38)
Session 2	81.4 (57)	
a: explanation about exposure and helplessness?		91.2 (52)
b: motivation to change discussed?		98.2 (56)
c: registration daily structure discussed?		94.7 (54)
Session 3	74.3 (52)	
a: instructions registration activities, anxiety, depression discussed?		96.2 (50)
b: partner/close relative invited?		78.8 (41)
Session 4	74.3 (52)	
a: consequence of symptoms on daily life discussed?		92.3 (48)
Session 5	71.4 (50)	
a: problem analysis made?		78.0 (39)
b: treatment goals specified?		84.0 (42)
Session 6	71.4 (50)	
a: explanation about attribution?		82.0 (41)
Session 7	65.7 (46)	
a: plan of activation made?		89.1 (41)
Session 8	62.9 (44)	
a: relapse prevention plan made?		75.0 (33)
Session 9	58.6 (41)	
a: crisis/signalling plan made?		82.9 (34)
Session10	58.6 (41)	
a: sleep-wake rhythm discussed?		92.7 (38)
Session 11	58.6 (41)	
a: dismissal date discussed?		90.2 (37)
b: desired care after intervention discussed?		85.4 (35)
c: contact with general practitioner?		46.3 (19)
Session 12	58.6 (41)	
a: symptom severity established?		68.3 (28)
b: appointments with other caregivers made?		58.5 (24)
Session 13	58.6 (41)	
a: final report made?		75.6 (31)
b: treatment completed?		80.5 (33)

## Discussion

### Main findings

This trial evaluated the effectiveness of a rehabilitation and self-management training for patients with chronic and treatment resistant anxiety and depressive disorders on quality of life, symptom severity, and empowerment. The results at 18-month follow-up regarding our primary outcome, quality of life measured with the WHOQOL-BREF, showed no significant differences between the experimental treatment and care as usual, except on the ‘social relationships’-domain (*P* = 0.041), with a moderate standardized effect size (*d* = 0.37) in favour of the ZemCAD group. There were no significant differences for anxiety symptom severity (BAI) and depressive symptom severity (PHQ-9) either. With regard to empowerment a significant difference between both groups was observed in the total empowerment (NEL) score and one empowerment dimension (caring community) of medium sizes (*d* = 0.45 and *d* = 0.39, respectively), both in favour of the ZemCAD group. Treatment integrity was assessed with a checklist that the trained professionals (mostly community psychiatric nurses) completed at the end of each treatment session. In total 19 (41.4%) of the patients did not finish all sessions of the ZemCAD intervention. After the ZemCAD intervention, more patients went from specialized outpatient mental health services back to a less specialized health care setting with less intensive treatment, such as primary care.

### Comparison with other studies

Only a few studies in this particular patient group are available for comparison. There are several self-management programs for patients with chronic psychiatric conditions that produce positive changes in health outcomes by teaching skills to better manage symptoms, enhance quality of life, and maintain higher levels of health and functioning. We found three of such programs, which are the Illness Management and Recovery (IMR) program, the Wellness Recovery Action Planning (WRAP), and the ‘Organized Self-Management Support Services’ all for patients with a broad range of mental disorders. In these programs positive effects were found on various outcomes. In comparison to previous studies that evaluated self-management programs in (chronic) psychiatric conditions in general, the current study was aimed specifically at patients with chronic and treatment resistant anxiety and depressive disorders. Hasson-Ohayon et al. [37] included patients with severe mental illness in general who received a more intensive treatment, while Cook et al. [38] focused on patients with anxiety and depression, but not a treatment resistant sample. The follow-up period was longer in the ZemCAD intervention. Ludman et al. [39] focused on patients with chronic depression, but had a longer, more intensive treatment than the ZemCAD intervention. All three studies had group settings and a peer support component was present. There was a focus on (symptomatic) recovery in all three studies as well contrary to our research, where the aim was on quality of life and empowerment.

### Findings in context

Given the long-term nature of anxiety and depressive symptoms in this group (the mean duration of current symptoms was 4.79 ± 7.14 years), we did not expect immediate symptomatic improvement. However, we did measure symptom severity to make sure that patients would not deteriorate, and found that patients using the ZemCAD intervention remained at least stable despite the fact that they knew that treatment in specialized mental health care would stop when they completed the ZemCAD intervention. Positive effects were expected in both quality of life and empowerment, and mainly found in the latter. Also on the ‘social relationships’-domain of the quality of life questionnaire a significant effect was found. The effects may have been too small to have had a substantial impact on quality of life, which is known to be a composite measure of satisfaction with personal functioning, self-esteem and many other things. Perhaps the way forward for patients with chronic and treatment resistant anxiety and depressive disorders is to accept the chronic nature of their disorder, and learn to develop effective self-management strategies [40] to enhance a shift from symptomatic remission towards improved personal and social functioning. It is unclear which interventions promote empowerment, but it is an assumption that self-management plays a major role [41]. Our study population had severe and chronic anxiety and depressive symptoms. They had a low quality of life, with scores below the average of the general population in the psychological and social domain [29]. Also there is a large number of low-educated patients (around 50%), and we know that in that case the possibilities for change are small [10]. The fact that there is a significant increase in empowerment in these patients is therefore promising. Whether the ZemCAD intervention offered the right approach to improve self-management and personal and social functioning, is questionable. With regard to the treatment integrity, we found that 19 (41.4%) of the patients did not finish all sessions of the ZemCAD intervention. This raises the question whether a higher compliance could lead to more positive effects on the outcome measurements of the ZemCAD intervention. Ending treatment in specialized mental health services was part of the ZemCAD intervention. This has not been successful in all cases, 44.0% was still receiving treatment in specialized outpatient mental health care, due to the high level of symptoms. This can also be related to compliance to the ZemCAD intervention.

### Strengths and limitations

This trial was conducted in a complex group of patients with chronic and treatment resistant anxiety or depressive disorders. We were able to include patients from various specialized mental health services in the Netherlands. Most patients maintained in the trial and completed the last measurements (78,8%). Despite the fact that patients were told in advance that treatment in specialized mental health services would stop after completing the ZemCAD intervention, we were able to include 141 patients with severe mental health problems. There were a number of limitations to this study. First, we used broad eligibility criteria. The inclusion criteria were based on the multidisciplinary guidelines and were defined in consultation with experts. To avoid being too restrictive and denying patients who might benefit from the intervention access to ZemCAD, we included patients that according to the MINI interview did not have an actual diagnosis, but were according to their health care provider eligible due to a high level of symptoms and a lifetime diagnosis. Furthermore it was possible to include patients that, for example, did not want to take a specific medication step like lithium addition. Not all patients in our study had therefore gone through the entire multidisciplinary guideline for the treatment of depression or anxiety. Although a disadvantage of this approach is that a fairly heterogeneous group of patients has been included, on the other hand this approach fits well with current practice, where it is not always possible to treat patients completely in accordance with the multidisciplinary guidelines. Second, we did not describe (co-morbid) personality disorders or social problems. These factors may also contribute to the chronicity of anxiety and depressive disorders. Third, the study relied on the participants’ self-reported data that were uncorroborated by clinicians or independent assessors. This may be a limitation, but provides information from the patients’ perspective instead of a clinical judgement. We know that clinicians are likely to be more positive when it comes to recovery progress. Fourth, although this study was not aiming to reduce symptoms, there was a lack of symptom measures. In addition to the participants’ self-reported symptom severity data (BAI and PHQ-9) we could have added, for example, the Hamilton Depression Rating Scale (HAM-D) and the Hospital Anxiety and Depression Scale – Anxiety subscale (HADS-A) for anxiety and depression symptom severity for more depth. Fifth, a significant proportion (47%) of the patients who were eligible to participate in the ZemCAD study did not want to participate. Patients mentioned several reasons. It is possible that those who were not included had more serious or chronic symptoms than the group that did participate.

## Conclusions

This trial on the effectiveness of a rehabilitation and self-management training for patients with chronic and treatment resistant anxiety and depressive disorders showed little effectiveness on a couple of measures between the experimental treatment and care as usual. However, patients remained at least stable, and in the ZemCAD group more patients (56% in the ZemCAD group against 38% in the CAU group) could be referred to less specialized mental health services, such as primary care. Since little is known about the effects of rehabilitation and self-management in patients with chronic and treatment resistant anxiety and depressive disorders on quality of life, symptom severity, and empowerment, this is a first attempt to provide a proof-of-concept study in this under-researched but important field.

### Directions for further research

The ZemCAD intervention did not aim at symptom reduction. Perhaps this group of patients with chronic and treatment resistant anxiety or depressive disorders needs more focus on symptom reduction, combined with self-management, to experience a higher quality of life. If we look at how effects developed over time (Table 3 and Appendix 2), we found significant differences in several outcomes in quality of life and empowerment, on the 6-month and 12-month follow-up measurement. When these effects occurred, they did not persist until the 18-month follow-up measurement. This could indicate that perhaps timely booster-sessions are required to sustain the positive effects in quality of life and empowerment. Further development of the ZemCAD intervention could involve a group program and the use of peer support. Groups enable patients to better withstand alienation and crisis because of the connectedness and social support [42]. Peer support is proven to be supplementary in various self-management programs [38, 39, 43]. Interactions with peers may enhance patients’ belief in their own ability to self-manage their lives, thus increasing perceptions of recovery and lowering feelings of anxiety and depression. It would be interesting to know what self-management strategies patients use to cope with a chronic and treatment resistant anxiety or depressive disorder, from the patients’ perspective. And it is relevant to look at the health gains of the ZemCAD intervention, from an economic point of view, relative to usual care.

## Appendix 1

**Table 5:** Estimated marginal means (EMM) of the outcomes

Outcome		ZemCAD (*n* = 70)	Control (*n* = 71)
		EMM (SEM^a^)	EMM (SEM)
Quality of life (WHOQOL-BREF^b^)			
Total score	Baseline	73.31 (1.73)	72.38 (1.75)
	6 months (T1)	74.89 (1.74)	73.27 (1.79)
	12 months (T2)	78.21 (1.78)	73.39 (1.82)
	18 months (T3)	77.36 (1.79)	74.54 (1.82)
Domain 1;	Baseline	18.13 (0.57)	17.86 (0.58)
Physical health	6 months (T1)	18.79 (0.58)	18.11 (0.60)
	12 months (T2)	20.40 (0.60)	18.30 (0.61)
	18 months (T3)	20.02 (0.61)	19.07 (0.61)
Domain 2;	Baseline	15.31 (0.43)	15.40 (0.43)
Psychological	6 months (T1)	15.80 (0.43)	15.41 (0.45)
	12 months (T2)	16.37 (0.45)	15.60 (0.46)
	18 months (T3)	16.34 (0.45)	15.62 (0.46)
Domain 3;	Baseline	8.23 (0.29)	8.62 (0.29)
Social relationships	6 months (T1)	8.55 (0.29)	8.77 (0.30)
	12 months (T2)	8.70 (0.30)	8.36 (0.31)
	18 months (T3)	8.66 (0.31)	8.60 (0.31)
Domain 4;	Baseline	26.56 (0.61)	25.60 (0.61)
Environment	6 months (T1)	26.67 (0.61)	26.09 (0.63)
	12 months (T2)	27.39 (0.63)	26.45 (0.64)
	18 months (T3)	27.15 (0.63)	26.21 (0.64)
Anxiety (BAI^c^)			
Total score	Baseline	43.76 (1.50)	45.42 (1.52)
	6 months (T1)	43.88 (1.52)	44.66 (1.56)
	12 months (T2)	40.88 (1.56)	43.84 (1.61)
	18 months (T3)	42.53 (1.58)	44.46 (1.60)
Depression (PHQ-9^d^)			
Total score	Baseline	22.46 (0.77)	23.33 (0.78)
	6 months (T1)	21.16 (0.78)	21.93 (0.80)
	12 months (T2)	19.86 (0.80)	20.63 (0.83)
	18 months (T3)	21.27 (0.82)	20.99 (0.83)
Empowerment (NEL^e^)			
Total score	Baseline	121.94 (2.35)	121.37 (2.38)
	6 months (T1)	125.58 (2.38)	119.59 (2.45)
	12 months (T2)	126.88 (2.44)	119.92 (2.50)
	18 months (T3)	123.66 (2.47)	123.08 (2.50)
Dimension 1;	Baseline	13.40 (0.57)	13.80 (0.58)
Professional help	6 months (T1)	13.65 (0.59)	12.78 (0.61)
	12 months (T2)	12.28 (0.62)	12.16 (0.63)
	18 months (T3)	9.39 (0.63)	12.23 (0.63)
Dimension 2;	Baseline	23.03 (0.58)	23.78 (0.59)
Social support	6 months (T1)	23.55 (0.59)	23.52 (0.61)
	12 months (T2)	24.10 (0.61)	23.77 (0.62)
	18 months (T3)	23.99 (0.61)	23.47 (0.62)
Dimension 3;	Baseline	34.98 (0.93)	34.29 (0.94)
Confidence and purpose	6 months (T1)	36.77 (0.94)	34.20 (0.96)
	12 months (T2)	37.63 (0.96)	34.86 (0.99)
	18 months (T3)	37.68 (0.97)	36.19 (0.99)
Dimension 4;	Baseline	18.65 (0.46)	18.20 (0.47)
Connectedness	6 months (T1)	19.41 (0.47)	17.77 (0.48)
	12 months (T2)	19.42 (0.49)	18.26 (0.50)
	18 months (T3)	19.27 (0.49)	18.94 (0.50)
Dimension 5;	Baseline	15.22 (0.37)	15.53 (0.38)
Self-management	6 months (T1)	15.48 (0.38)	15.72 (0.39)
	12 months (T2)	16.15 (0.39)	15.48 (0.40)
	18 months (T3)	16.20 (0.40)	16.03 (0.40)
Dimension 6;	Baseline	16.66 (0.47)	15.79 (0.47)
Caring community	6 months (T1)	16.84 (0.48)	15.67 (0.49)
	12 months (T2)	17.51 (0.49)	15.45 (0.50)
	18 months (T3)	17.29 (0.50)	16.27 (0.50)

^a^*SEM* Standard error of the mean

^b^*WHOQOL-BREF* World Health Organisation Quality of Life, Brief version

^c^*BAI* Beck Anxiety Inventory

^d^*PHQ-9* Patient Health Questionnaire

^e^*NEL* Netherlands Empowerment List

## Appendix 2

**Table 6:** Estimated differences^a^ in mean change at 6, 12 and 18 months

Outcome		Estimate of mean difference (95% CI)	*t (df)*	*P*
Quality of life (WHOQOL-BREF^b^)				
Total score	6 months (T1)	0.69 (− 2.56 to 3.93)	0.42 (354)	0.678
	12 months (T2)	3.88 (0.49 to 7.27)	2.25 (356)	0.025*
	18 months (T3)	1.89 (− 1.53 to 5.31)	1.09 (356)	0.279
Domain 1;	6 months (T1)	0.41 (− 0.94 to 1.76)	0.60 (357)	0.549
Physical health	12 months (T2)	1.82 (0.42 to 3.23)	2.55 (361)	0.011*
	18 months (T3)	0.68 (− 0.74 to 2.10)	0.95 (361)	0.345
Domain 2;	6 months (T1)	0.48 (− 0.43 to 1.40)	1.04 (354)	0.300
Psychological	12 months (T2)	0.87 (− 0.08 to 1.83)	1.79 (357)	0.074
	18 months (T3)	0.82 (− 0.15 to 1.78)	1.66 (357)	0.098
Domain 3;	6 months (T1)	0.17 (− 0.46 to 0.79)	0.52 (353)	0.603
Social relationships	12 months (T2)	0.72 (0.07 to 1.37)	2.18 (356)	0.030*
	18 months (T3)	0.44 (− 0.21 to 1.10)	1.33 (356)	0.186
Domain 4;	6 months (T1)	−0.37 (− 1.61 to 0.87)	−0.59 (355)	0.556
Environment	12 months (T2)	−0.01 (− 1.30 to 1.29)	−0.01 (357)	0.993
	18 months (T3)	−0.01 (− 1.32 to 1.30)	−0.02 (357)	0.986
Anxiety (BAI^c^)				
Total score	6 months (T1)	0.88 (− 2.37 to 4.12)	0.53 (352)	0.595
	12 months (T2)	−1.31 (− 4.72 to 2.10)	−0.76 (355)	0.451
	18 months (T3)	−0.27 (− 3.70 to 3.16)	−0.16 (355)	0.875
Depression (PHQ-9^d^)				
Total score	6 months (T1)	0.09 (− 1.74 to 1.92)	0.10 (354)	0.921
	12 months (T2)	0.09 (−1.82 to 2.01)	0.10 (358)	0.923
	18 months (T3)	1.14 (− 0.79 to 3.08)	1.16 (358)	0.247
Empowerment (NEL^e^)				
Total score	6 months (T1)	5.41 (0.42 to 10.40)	2.13 (353)	0.034*
	12 months (T2)	6.39 (1.19 to 11.59)	2.42 (355)	0.016*
	18 months (T3)	0.00 (− 5.24 to 5.25)	0.00 (355)	0.999
Dimension 1;	6 months (T1)	1.26 (− 0.55 to 3.08)	1.37 (354)	0.172
Professional help	12 months (T2)	0.52 (−1.37 to 2.40)	0.54 (360)	0.590
	18 months (T3)	−2.45 (− 4.35 to − 0.55)	−2.53 (361)	0.012*
Dimension 2;	6 months (T1)	0.78 (− 0.54 to 2.09)	1.16 (353)	0.245
Social support	12 months (T2)	1.07 (− 0.30 to 2.43)	1.54 (356)	0.125
	18 months (T3)	1.27 (− 0.11 to 2.65)	1.82 (356)	0.070
Dimension 3;	6 months (T1)	1.88 (− 0.12 to 3.88)	1.84 (354)	0.066
Confidence and purpose	12 months (T2)	2.08 (− 0.01 to 4.16)	1.96 (356)	0.051
	18 months (T3)	0.80 (−1.30 to 2.91)	0.75 (356)	0.452
Dimension 4;	6 months (T1)	1.19 (0.08 to 2.31)	2.11 (355)	0.036*
Connectedness	12 months (T2)	0.71 (− 0.44 to 1.87)	1.21 (358)	0.226
	18 months (T3)	−0.11 (− 1.28 to 1.06)	−0.18 (358)	0.857
Dimension 5;	6 months (T1)	0.07 (− 0.85 to 1.00)	0.15 (354)	0.878
Self-management	12 months (T2)	0.98 (0.02 to 1.95)	2.01 (357)	0.045*
	18 months (T3)	0.48 (−0.49 to 1.48)	0.97 (358)	0.332
Dimension 6;	6 months (T1)	0.30 (− 0.83 to 1.42)	0.52 (355)	0.604
Caring community	12 months (T2)	1.19 (0.02 to 2.36)	2.00 (358)	0.046*
	18 months (T3)	0.15 (− 1.03 to 1.33)	0.25 (359)	0.802

*Significant difference

^a^All estimates are adjusted for ‘partner status’ and ‘duration of current symptoms’

^b^*WHOQOL-BREF* World Health Organisation Quality of Life, Brief version

^c^*BAI* Beck Anxiety Inventory

^d^*PHQ-9* Patient Health Questionnaire

^e^*NEL* Netherlands Empowerment List

## Abbreviations

BAI: Beck anxiety inventory
CAU: Care as usual
HADS-A: Hospital Anxiety and Depression Scale – Anxiety subscale
HAM-D: Hamilton Depression Rating Scale
IMR: Illness Management and Recovery
ITT: Intention-to-treat
LMM: Linear mixed model analysis
MINI interview: Mini-International Neuropsychiatric Interview
NEL: Netherlands Empowerment List
NEMESIS-2: Netherlands Mental Health Survey and Incidence Study-2
PHQ-9: Patient Health Questionnaire
REML: Residual maximum likelihood
WHOQOL-BREF: World Health Organization quality of life instrument brief version
WRAP: Wellness Recovery Action Planning
ZemCAD (English SemCAD): Self-management for Chronic Anxiety and Depression
